# Pre-sleep casein protein ingestion: new paradigm in post-exercise recovery nutrition

**DOI:** 10.20463/pan.2020.0009

**Published:** 2020-06-30

**Authors:** Jooyoung Kim

**Affiliations:** 1 Office of Academic Affairs, Konkuk University, Chungju Republic of Korea

**Keywords:** casein protein, post-exercise recovery, protein balance, protein synthesis, sleep

## Abstract

**[Purpose]:**

Milk is a commonly ingested post-exercise recovery protein source. Casein protein, found in milk, is characterized by its slow digestion and absorption. Recently, several studies have been conducted with a focus on how pre-sleep casein protein intake could affect post-exercise recovery but our knowledge of the subject remains limited. This review aimed at presenting and discussing how pre-sleep casein protein ingestion affects post-exercise recovery and the details of its potential effector mechanisms.

**[Methods]:**

We systematically reviewed the topics of 1) casein nutritional characteristics, 2) pre-sleep casein protein effects on post-exercise recovery, and 3) potential effector mechanisms of pre-sleep casein protein on post-exercise recovery, based on the currently available published studies on pre-sleep casein protein ingestion.

**[Results]:**

Studies have shown that pre-sleep casein protein ingestion (timing: 30 minutes before sleep, amount of casein protein ingested: 40-48 g) could help post-exercise recovery and positively affect acute protein metabolism and exercise performance. In addition, studies have suggested that repeated pre-sleep casein protein ingestion for post-exercise recovery over a long period might also result in chronic effects that optimize intramuscular physiological adaptation (muscle strength and muscle hypertrophy). The potential mechanisms of pre-sleep casein protein ingestion that contribute to these effects include the following: 1) significantly increasing plasma amino acid availability during sleep, thereby increasing protein synthesis, inhibiting protein breakdown, and achieving a positive protein balance; and 2) weakening exercise-induced muscle damage or inflammatory responses, causing reduced muscle soreness. Future studies should focus on completely elucidating these potential mechanisms.

**[Conclusion]:**

In conclusion, post-exercise ingestion of at least 40 g of casein protein, approximately 30 minutes before sleep and after a bout of resistance exercise in the evening, might be an effective nutritional intervention to facilitate muscle recovery.

## INTRODUCTION

Post-exercise recovery is a topic of considerable interest in the field of exercise nutrition. In particular, protein intake has been considered very important in nutritional strategies for muscle recovery^1^. This is because while exercise improves the physiological adaptation of skeletal muscle, protein breakdown is increased due to muscle damage and inflammation after exercise^2^. Sufficient protein intake could contribute to a positive protein balance during post-exercise recovery, reduce protein breakdown, and increase protein synthesis, a process through which amino acids produce peptides and proteins^3^. Through this process, protein turnover typically occurs and muscle remodeling is achieved. However, a normal daily protein intake alone might not be sufficient to increase protein synthesis and speed up recovery after exercise^4^. Delayed muscle recovery due to insufficient protein intake after sports, games, or resistance training in the evening has been reported^5^, thus raising the need for an additional protein intake before sleep.

Given the fact that no further nutritional intake occurs for several hours during sleep, pre-sleep casein protein intake has recently been recommended^5,6^. Casein protein accounts for a large portion of the total protein in milk and is characterized by its slow digestion and absorption^7^. Several studies in humans have reported that pre-sleep casein protein ingestion after exercise has positive effects on muscle recovery^5,8^. Hence, pre-sleep casein protein ingestion has been suggested as a new window of opportunity in nutrient timing^9^. Despite this trend, while there are many studies on the benefits and effects of whey protein in the field of exercise nutrition, studies on casein protein are limited. Therefore, this review aimed to present and discuss the effects of pre-sleep casein protein ingestion on post-exercise recovery and its potential mechanisms.

### Nutritional characteristics of casein protein

Milk is a common protein source ingested for muscle recovery. Milk contains the following two major types of protein: casein and whey^10,11^. Casein protein exists in the form of various micelles, and it is composed of alpha-s1, alpha-s2, and beta- and kappa-casein and is present in the highest proportion in milk, making up 75-80 % of all milk proteins^12^. Casein protein provides all essential amino acids to humans, except cysteine, and is classified as a high-quality protein source with high digestibility and bioavailability evaluated by indices such as the protein digestibility corrected AA score (PDCAAS) and digestible indispensable amino acid score (DIAAS)^13^. In contrast, whey protein has a higher proportion of leucine, isoleucine, and valine than casein protein. Additionally, non-essential amino acids such as arginine, glutamic acid, proline, serine, tyrosine, histidine, methionine, and phenylalanine are more abundant in casein protein^14^.

When casein protein is in low pH conditions (acidification), such as pH = 4.6, the repulsive force of anions between the molecules disappears, allowing them to bind to each other, causing precipitation. This reduces the gastric emptying rate, which slows the digestion and absorption of casein protein, thereby delaying its breakdown and flow into the blood amino acid pool^12^. Therefore, an increase in plasma amino acid concentration after casein protein ingestion occurs more slowly compared to whey protein. Consequently, casein protein is called “slow” protein, and whey protein is called “fast” protein^1^. In fact, according to the results of a recent study, the ingestion of whey or casein protein after exercise showed different peak time patterns in increasing muscle protein synthesis rates, in which whey protein peaked 60 minutes after exercise and casein protein peaked 120 minutes after exercise^10^. Another study reported that muscle protein synthesis was increased for 3.5 hours after whey protein ingestion, whereas muscle protein synthesis was increased for up to 6 hours after casein protein ingestion^15^. In addition, protein anabolism after exercise was reported to be better maintained after the ingestion of casein protein compared to whey protein^16^.

### Effects of pre-sleep casein protein on post-exercise recovery 

Several studies have reported that pre-sleep casein protein intake could have positive effects on post-exercise recovery. A study involving healthy young men showed that 40 g of casein protein ingested 30 minutes before sleep following resistance training was digested and absorbed well during sleep. Additionally, their circulating amino acid levels increased rapidly, resulting in increased whole-body protein synthesis rates and improved protein balance, inducing positive effects on muscle recovery^8^. In addition, a study involving active women who ingested casein protein at either a low (24 g) or high dose (48 g) found no statistically significant differences but found that the volume of resistance training they could perform the next day was slightly increased after ingesting 48 g casein protein 30 minutes before sleep. Meanwhile, the ingestion of a low dose of casein protein (24 g) did not affect resistance training volume the next day^9^.

A recent study involving English soccer players found that the ingestion of 40 g of casein protein 30 minutes before sleep after a competitive match had positive effects on counter-movement jump recovery and reactive strength index recovery at 12 and 36 hours after the match. Further, muscle soreness measured by the visual analog scale (VAS) was also significantly reduced 12 hours after the match compared to the control group5. Soccer players often do not eat adequately for recovery after a match or training performed in the evening, or they often do not ingest enough protein. This nutrition-related problem could delay recovery because it could lead to a protein imbalance in the body, reducing protein synthesis rates during sleep^17^. Therefore, if soccer players consider pre-sleep casein protein intake after an evening match or training, they will be able to prepare for the next game or training with less fear of performance deficits.

Eventually, stimulating post-exercise recovery by pre-sleep casein protein ingestion will have a significant impact on achieving ultimate fitness goals from a long-term perspective. This is because post-exercise recovery is the underlying factor for optimizing muscle physiological adaptation (muscle strength, and muscle hypertrophy) after exercise^18^. Acute changes in muscle protein synthesis and degradation rates after exercise could predict adaptive responses to more prolonged interventions^19^. Several experimental studies have reported that muscle strength and muscle hypertrophy are significantly improved after a combination of regular resistance exercise and pre-sleep casein protein ingestion for a long period (e.g., more than 10 weeks)^6,20,21^. The results of these studies are evidence that when repeated over time, pre-sleep casein protein ingestion and the resulting muscle recovery could effectively result in chronic positive effects on muscle adaptation.

Meanwhile, unlike the aforementioned positive findings, some studies have reported that pre-sleep casein protein ingestion did not affect post-exercise recovery. A study of young males found that ingesting 30 g of casein protein 30 minutes before sleep following a bout of resistance training had no significant effects on myofibrillar protein synthesis rates, regardless of the addition of 2g of leucine^22^. A recent study also reported that intramuscular connective tissue protein synthesis rates were not significantly increased in healthy young males who ingested 30 g of casein protein 30 minutes before sleep following resistance exercise, compared to those who ingested a placebo^23^. A possible reason for these negative results could be the amount of casein protein ingested as 40-48 g of casein protein had positive effects in previous studies^8,9^, whereas only 30 g of casein protein was ingested in the studies showing no effects^22,23^. However, a recent study involving active males and females reported that the ingestion of 40 g of casein protein 30 minutes before sleep after repeated high-intensity drop jumps did not affect acute functional recovery indices such as countermovement jumps or pressure-pain threshold^2^, indicating that the results of the previous studies might not be due to the amount of casein protein ingested alone. Perhaps these differences might be due to the type of exercise performed or its timing (morning or evening). In a study conducted by Apweiler et al.^2^, drop jumps were performed in the morning, whereas, in the other previous studies, casein protein was ingested after performing resistance training in the evening^8,9,21^. The time of the day that the exercise is performed might be considered as a potential factor influencing the results of this study because the anabolic response might slow down due to subsequent activities even after the ingestion of casein protein following the morning exercise. Further research is required to support this idea; however, we believe that it will be different from when you sleep in a stable state after ingesting casein protein. These conflicting findings suggest that further studies regarding pre-sleep casein protein are needed. However, when comprehensively reviewing the studies published to date, the consensus is that pre-sleep casein protein ingestion, especially after resistance training in the evening, could have positive effects on muscle recovery.

### Potential mechanisms of pre-sleep casein protein on post-exercise recovery

Several studies have reported that pre-sleep casein protein ingestion was well digested and absorbed during overnight sleep, thereby significantly increasing the amino acid content in the blood^8,24^. Phenylalanine concentrations in blood were reported to be significantly increased during overnight recovery after pre-sleep casein protein ingestion8. Further, the ingestion of 40 g of pre-sleep casein protein increased plasma amino acid availability more significantly over a long period compared to the ingestion of 20 g of pre-sleep casein protein^24^. These changes are known to cause “hyperaminoacidemia,” a condition characterized by high amino acid content in the blood^25^. Hyperaminoacidemia caused by dietary protein intake increases the gene expression of several amino acid transport proteins, and thus increases penetration of amino acids into the muscle membrane^26^. As a result, this increases amino acid influx to the skeletal muscle, and these changes regulate the relative balance between protein synthesis and protein breakdown. This is an important factor that increases protein synthesis, inhibits protein breakdown, and enables a positive protein balance during the overnight period^27,28^. Such changes in protein metabolism seem to act as the biggest contributor to stimulating post-exercise recovery. Further studies on the molecular mechanisms that determine protein metabolism-related changes are needed in the future to explain protein synthesis during the overnight period after pre-sleep casein protein ingestion and its muscle recovery mechanism.

Another possibility is that the positive protein balance induced by pre-sleep casein protein ingestion stimulates muscle recovery while reducing exercise-induced muscle damage. In general, muscle damage causes reduced muscle function, increased muscle soreness, and a mild inflammatory response^29^. Pre-sleep casein protein ingestion could mitigate the inflammatory response and speed up the muscle remodeling process. In a study by Abbott et al.^5^, muscle soreness was reported to be significantly reduced after pre-sleep casein protein ingestion. Although studies of muscle soreness are controversial, it is one of the indirect pieces of evidence of an inflammatory response^30^. However, to the best of our knowledge, there are no studies directly reporting a decreased inflammatory response after pre-sleep casein protein ingestion.

**Figure 1.**
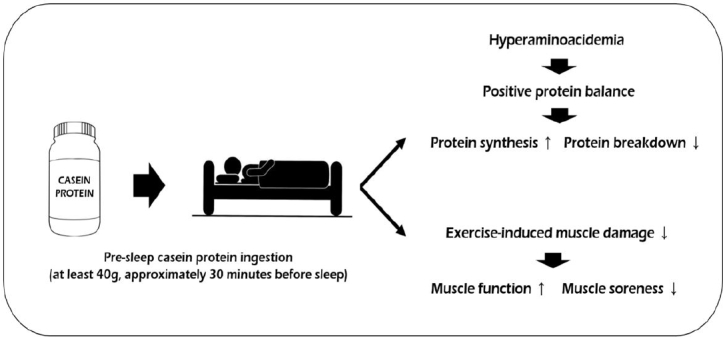
Potential mechanisms of pre-sleep casein protein on post-exercise recovery

In summary, the mechanisms through which exerts positive effects on post-exercise recovery might include the following (Figure 1): 1) achieving a positive protein balance by increasing plasma amino acid availability, and 2) reducing muscle damage and inflammatory response. However, further well-organized studies examining these potential mechanisms are needed in the future.

## CONCLUSION

In conclusion, post-exercise ingestion of at least 40 g casein protein 30 minutes before sleep, especially after resistance exercise in the evening, could be an effective nutritional intervention to stimulate muscle recovery. However, several studies report age-dependent differences in the effect or response pattern after pre-sleep casein protein ingestion (e.g., young adults compared to the elderly), which should be considered when ingesting casein protein^31^. Further future studies should focus on pre-sleep casein protein ingestion and its potential mechanisms affecting recovery. In particular, studies investigating changes in recovery after casein ingestion following endurance exercise would be warranted.

